# The left temporal transverse cortex is affected by poor sleep quality, which in turn contributes to depressive symptoms in older adults^☆^

**DOI:** 10.1016/j.heliyon.2023.e20751

**Published:** 2023-10-07

**Authors:** Wei Li, Lin Sun, Ling Yue, Shifu Xiao

**Affiliations:** a Department of Geriatric Psychiatry, Shanghai Mental Health Center, Shanghai Jiao Tong University School of Medicine, Shanghai, China; b Alzheimer's Disease and Related Disorders Center, Shanghai Jiao Tong University, Shanghai, China

**Keywords:** Sleep quality, Anxiety, Depression, Cortex thickness, Transverse temporal gyrus

## Abstract

Sleep quality is critical for improving mental health among older adults. Despite this, there is a dearth of studies examining the correlation between sleep quality and emotional symptoms in the elderly population of China. This study included 496 community elders aged 55 years and older. The participants were divided into two groups based on their scores on the Pittsburgh Sleep Quality Index (PSQI), with 249 being classified as poor sleepers and 247 as good sleepers. All participants were asked to fill out a uniform survey which included details about their demographics, daily habits, and any illnesses they were dealing with. The Self-rating anxiety scale (SAS) and Geriatric Depression Scale (GDS) were employed to measure their levels of anxiety and depression, respectively. In addition, 50 healthy individuals also agreed to brain MR imaging. The finding of our study indicated that those with inadequate sleep had higher levels of depression and anxiety, and the overall anxiety and depression score was linked to the total PSQI score in a positive manner; The MRI subgroup analysis revealed that those with inadequate sleep quality had a greater thickness of the left transverse temporal gyrus (p < 0.05). In addition, a Linear regression analysis of the mediation model showed that poor sleep quality would result in higher scores on the GDS, and cortical thickness in the left transverse temporal gyrus played a fully mediated role in this process. Our research indicates that elderly people in community who have difficulty sleeping may be more likely to suffer from anxiety and depression, and this lack of sleep can result in depressive symptoms due to its impact on the thickness of the left transverse temporal cortex.

## 1 Introduction

Epidemiological evidence indicates that individuals aged 65 and above frequently experience a range of sleep disorders, including insomnia, snoring, narcolepsy, periodic leg movement disorder, restless legs syndrome, and rapid eye movement (REM) sleep behavior disorder [1].

These sleep problems not only affect the quality of life for adults, but can also lead to the development of other diseases, such as pain, asthma, diabetes, dementia, hypertension, immune dysfunction, physical disability, gastro-esophageal reflux disease, and mental illness [2]. In addition, a lack of restful sleep can lead to a decline in mental acuity among elderly people, thus increasing the likelihood of sports and other motor vehicle collisions, industrial mishaps, medical mistakes, and a decrease in productivity in the workplace [3].

Examining the elements that have an effect on sleep quality will assist us in formulating more effective preventive strategies, and prior research has uncovered a variety of elements that may lead to inadequate sleep quality, such as diabetes [4], menopausal syndrome [5], vitamin D deficiency [6], overweight/obese [7], not being married, having a low personal income [8], being female and living in a rural area [9]. In addition, some articles also pointed that mental health disorders could predict decreased sleep quality. An example of this is Wu CY et al.s’ research, which revealed that depression was the only element that had a noteworthy correlation with inadequate sleep quality after adjustment [10]. Ding L et al.’s research revealed that the amount and standard of sleep can have an effect on the psychological wellbeing of elderly women [11]. Xiao S. et al.’s research revealed that anxiety and depression symptoms had an impact on the cognitive performance of elderly individuals when it came to their subjective sleep quality [12]. Hu Z et al.’ s research suggested that inadequate sleep may be a sign of depression in the elderly [13]. Guo H et al.’s research revealed that the effect of cognition on depression was partially attributed to its effect on sleep quality [14]. Furthermore Vazquez-Delgado E et al.’ s research revealed that psychological distress was significantly linked to sleep quality, regardless of age, gender, length of time, medication use, and pain intensity [15].

Imaging analysis is one of the most effective tools for studying brain structure and function, and it is widely used in both clinical and scientific research institutions. Most of the current literature, however, has only described this phenomenon, with only a few studies exploring possible imaging mechanisms by which affective symptoms affect sleep quality. For example, Cheng W et al. found that a functional connection in the brain (including angular gyrus, precuneus, cingulate cortex, temporal cortex and lateral orbitofrontal cortex) may be mediating the association between problems with depression and sleep quality [16]. In the research conducted by Toschi N et al., they found a correlation between inadequate sleep quality and brief sleep duration and a decrease in intracortical myelin in the middle posterior cingulate cortex, middle temporal cortex, and anterior orbitofrontal cortex [17]. Guadagni V et al.'s study found that the quality of one's sleep was associated with emotional empathy responses by increasing neural activation in specific areas of the insular cortex [18]. In addition, Zhang L et al.'s study found that negative emotions have a common brain structure and function as a function of sleep quality [19].

The superior temporal gyrus encompasses the transverse temporal gyrus, alternatively referred to as the Heschl gyrus or Heschl cyclotron. The lower two thirds of the primary auditory cortex, as well as the anterolateral subregion, which is essential for pitch processing, are both located in this area [20]. The transverse temporal gyrus may play a significant regulatory role in the relationship between sleep quality and mood symptoms, according to a growing body of research. The research conducted by Zhang L et al. revealed a strong correlation between sleep quality and psychological stress, with gray matter volume in the bilateral inferior temporal gyrus being a major factor in improving sleep quality and emotional processing [21]. In Kong L et al.’s study, it was found that patients with obstructive sleep apnea (OSA) exhibited disturbed functional connectivity (FC) from the dorsal anterior insula (dAI) to areas relevant to cognition, like the temporal gyrus, in comparison to the healthy control group [22]. Furthermore, Wang Y et al.'s research revealed that the transverse temporal gyrus could be a factor in the effect of anxiety and depression on the quality of sleep in healthy people [23]. Consequently, we postulated that anxiety and depressive symptoms could potentially be linked to the quality of sleep, while the transverse temporal gyrus could potentially facilitate the correlation between mood and sleep quality.

In order to examine these findings, a total of 496 adults, all aged 55 years and above, were enlisted from the local community. The Pittsburgh Sleep Quality Scale was used to evaluate their sleep quality, while the self-rating Anxiety Scale (SAS) and the Geriatric Depression Scale (GDS) were employed to measure their anxiety and depression symptoms. A select few individuals also underwent magnetic resonance imaging concurrently. We hypothesized that elderly people in the community who had poorer sleep quality were more likely to suffer from anxiety and depression. It is conceivable that certain components of the brain, such as the transverse temporal gyrus, may have played a role in this process.

Currently, there are few studies on the relationship between sleep quality and mood disorders in older adults in the community, and the mechanisms are unclear. The current study fills a gap in research at home and abroad: not only can the relationship between sleep quality and emotional symptoms such as anxiety and depression be explored in detail, but it can also reveal possible imaging mechanisms of sleep disorders caused by mood disorders and provide relevant theoretical basis for future intervention and prevention strategies.

## 2 Methodology

### 2.1 Participants

The present cohort study was drawn from the Brain Health Cohort study conducted in Shanghai (http://www.shanghaibrainagingstudy.org/), which has been extensively documented in our prior research [ [24,25]]. The eligibility requirements included: 1) being at least 55 years old; 2) having all necessary information completed; 3) not having any significant mental illnesses like severe depression and schizophrenia; 4) not having any severe medical conditions such as infections or cancer. Exclusion criteria are: 1) less than 55 years of age; 2) drug or alcohol dependence abuse; 3) use of drugs that may interfere with sleep or mood; 4) lack of data. Finally, a total of 496 community-dwelling participants aged 55 or older were included in our analysis. All eligible participants were then asked to complete a standard questionnaire that included general information (e.g., age, education, gender), daily patterns (e.g., smoker drinking, tea drinker, exercise) and information about related diseases (e.g., hypertension, diabetes). They would be screened at the same time, including physical and neurologic examinations, sleep quality assessments and psychiatric assessments. In addition, those who consented were invited to participate in qualitative research [in-depth interviews or focus groups] and quantitative research. In order to explore the mechanism of mental status affecting sleep quality, 50 healthy individuals with no mild cognitive impairment and no dementia agreed to brain MR imaging (poor sleeper n = 21; good sleeper n = 29). All subjects were evaluated by experienced attending physicians.

The study was ethically approved by the Ethics Committee of Shanghai Mental Health Center, **the ethical approval number was 2018**–**11R,** and all participants signed their informed consent before starting the study. The whole study was based on the tenets of the Helsinki Declaration.

### 2.2 Pittsburgh Sleep Quality Index (PSQI)

This research employed the Pittsburgh Sleep Quality Index (PSQI) to assess the sleep quality of the participants. The PSQI, consisting of nineteen items, is a self-report questionnaire utilized to assess the subjective sleep quality during the previous month. The 19 items of the PSQI are combined into seven items to evaluate different facets of sleep, such as subjective sleep quality, sleep latency, sleep duration, sleep efficiency, sleep disturbance, sleep medication, and daytime dysfunction [26]. The PSQI score varies from 0 to 21, with a score of 5 or higher signifying inadequate sleep quality, while a score of less than 5 implies satisfactory sleep quality [27]. Numerous clinical studies have showed that PSQI has a high degree of sensitivity (89.6 %) and specificity (86.5) (kappa = 0.75, p < 0.001), allowing for a clear distinction between those with good sleep quality and those with poor sleep quality [ [26,28]]. Likewise, in our current study, we also used a score of 5 as a cutoff point between poor and good sleepers.

### 2.3 Geriatric Depression Scale (GDS) and self-rating anxiety scale (SAS)

The GDS and SAS were employed to gauge feelings of depression and anxiety in the elderly. The GDS-15 is a useful tool for classifying depression in older adults as mild or severe. A rating from 0 to 15 is assigned to the scale, and a score of 5 or higher is thought to be a sign of depression [29]. Like the GDS, the SAS is also a commonly used tool to assess anxiety symptoms in the elderly population [30]. SAS is a widely used gold standard scale for screening anxiety disorders. The score can range anywhere between 0 and 30 points. An anxiety symptom is usually indicated by a score of 10 or higher. Currently, a number of studies have confirmed that the SAS has good sensitivity and specificity, which can be applied in both clinical and experimental studies [31].

All of the above scales used by us were all translated versions, and the process of translating them into Chinese was as follows [ [32,33]]: interpretation, cross-cultural adjustment, trial run, and psychometric examination. Phase 1 interpretation: the forward translation process is done by two bilinguals (native Chinese speakers), and the reverse translation is done by two native English speakers who have not seen the original materials and tools. The research team then contrasted translations and deliberated on an appropriate draft translation. Phase 2 cross-cultural adjustment: To ascertain if the tools created in English could be applied to Chinese culture and to guarantee the accuracy of the tool measurements, face-to-face interviews were conducted with translated texts. Through convenient sampling, we selected 10 medical staff to investigate their understanding and opinions on various items of the translated questionnaire. Participants were selected as front-line clinical staff who were volunteered to participate in the study. Phase 3 trial run: The expert consultation questionnaire is broken down into three parts: (1) A letter to the experts: Introduction to the purpose and content of the study, background to the translation of the questionnaire; (2) Expert basic information table: demographic information of the experts, familiarity with the research issues and basis for judgement; (3) Expert evaluation table: Experts should be asked to choose the relevance or representativeness of each project in the translation tool in terms of the corresponding content, and should be divided into four levels (1 = irrelevant, 2 = relevant, 3 = relative, 4 = very relevant), as well as the proposed modifications to each project and overall recommendations of the study. Phase 4 psychometric examination: Researchers visited clinical departments, where doctors are first asked to fill out a paper questionnaires and asked if there were a program that were difficult to understand. If so, the researchers will interpret and document the problem project. All of the above scales were performed by psychometric evaluators, all of whom received consistent training prior to assessment.

### 2.4 The process of obtaining and analyzing magnetic resonance images

The Siemens Magnetom Verio 3.0 T scanner (Siemens, Munich, Germany) was responsible for obtaining these images, and we have extensively discussed them in our previous article. [ [34,35]]. To put it succinctly, 176 sagittal slices were used to obtain T1-weighted images with a 3D magnetization fast gradient echo acquisition sequence. The acquisition parameters were as follows: TR = 230 ms, TE = 2.98 ms, rotation Angle = 9°, spatial resolution = 1 * 1 * 1.2 mm^3^. All structural MRI data were clinically processed in FreeSurfer v6.0, including spatial registration, cortical thickness estimation, cortical surface segmentation extraction of subcortical structures, and segmentation sequences of 46 global structures [36]. Each individual's transverse temporal gyrus volume and thickness were then extracted directly using FreeSurfer.

### 2.5 The design of the entire study

The overall study design aimed to initially assess the sleep quality of the subjects using the PSQI followed by a comparison of anxiety and depression scores between the two groups in order to investigate the internal relationship between symptoms of anxiety and depression and sleep quality. Subsequently, structural magnetic resonance imaging was employed to further elucidate potential mechanisms through which symptoms of anxiety or depression impact sleep quality.

## 3 Data analysis

The demographic characteristics of the subjects were predominantly categorical variables, thus they were presented as frequencies and percentages. Meanwhile, anxiety and depression scores were expressed as mean ± standard deviation (SD). Overall sleep quality, assessed by the Global Sleep Quality Index (PSQI) score, was evaluated both number and categorically based on the severity of sleep difficulties (“good sleep” versus “poor sleep”). The PSQI value and its component scores in terms of mean (M), standard deviation), quartiles, and range. Categorical variables comparing poor sleep quality with good sleep quality were analyzed using a χ2 test, while continuous variables between the two groups were compared using an independent sample *t*-test. Furthermore, a binary logistic regression model was employed to examine the relationship between sleep quality and emotional symptoms. Correlation analysis was conducted to explore the association between PSQI scores and GDS and SAS scores. Finally, linear regression analysis was performed with GDS or SAS score as the dependent variable and left/right temporal gyrus thickness as the independent order to investigate the relationship between emotional scores and related to sleep. All statistical-tailed test with a significance level p < 0.05. Data analysis was conducted using SPSS 22.0 software (IBM Corporation, Armonk, NY).

## 4 Results

### 4.1 General subject demographics

**General subject demographics are presented in**Table 1. Of the 496 community elders, 191 were men, comprising 38.5% of the community. 17.1 % were in the 55–59-year arm, 24.0 % were in the 60–64-year arm, 26.2 % were in the 65–69-year arm, 17.7 % were in the 70–74-year arm, 8.5 % were in the 75–79-year arm, and 6.5 % were in the 80 years plus arm. 4.8 % were illiterate, 8.3 % had a primary school education, 46.6 % had a junior high school education, 30.6 % had a high school or technical secondary school education, 7.5 % had a junior college education, and 2.2 % had a university or above education. 105 (21.2 %) were smokers, 114 (23.0 %) were drinkers, 218 (44.0 %) were tea drinkers, 313 (63.1 %) had physical exercise, 219 (44.2 %) had hypertension, 77 (15.5 %) had diabetes. Their average anxiety score was 31.84 ± 6.74, and their average anxiety score was 2.84 ± 2.93. In addition, the average PSQI score of the elderly living in the community was 5.21 ± 3.79.

**Table 1:** Sample characteristics of the elderly in the community (n = 496).

Characteristics	N (%)
Gender	
Male	191 (38.5)
Female	305 (61.5)
Age	
55-59	85 (17.1)
60-64	119 (24.0)
65-69	130 (26.2)
70-74	88 (17.7)
75-79	42 (8.5)
80-	32 (6.5)
Educational level	
illiterate	24 (4.8)
Primary school	41 (8.3)
Junior high school	231 (46.6)
High school or technical secondary school	152 (30.6)
Junior college	37 (7.5)
University or above	11 (2.2)
Smoker	
Yes	105 (21.2)
No	391 (78.8)
Drinker	
Yes	114 (23.0)
No	382 (77.0)
Tea drinker	
Yes	218 (44.0)
No	278 (56.0)
Physical exercise	
Yes	313 (63.1)
No	183 (36.9)
Hypertension	
Yes	219 (44.2)
No	277 (55.8)
Diabetes	
Yes	77 (15.5)
No	419 (84.5)
SAS, mean ± SD	31.84 ± 6.74
GDS, mean ± SD	2.84 ± 2.93
PSQI component/Domain	
Subjective sleep quality	0.99 ± 0.78
Sleep latency	0.98 ± 1.03
Sleep duration	1.11 ± 0.95
Sleep efficiency	0.94 ± 1.20
Sleep disturbance	0.74 ± 0.54
Use of sleep medication	0.15 ± 0.56
Daytime dysfunction	0.30 ± 0.66
Global PSQI score	5.21 ± 3.79

Abbreviation: SAS, self-rating Anxiety Scale; GDS, Geriatric Depression Scale; PSQI, Pittsburgh Sleep Quality Index.

### 4.2 The association between affective symptoms and sleep quality

Relative to the good sleepers, the poor sleepers had higher SAS scores and higher GDS scores (p < 0.05) (Table 2). With sleep quality as the dependent variable, anxiety and depression as the independent variable, and age and gender as the covariates, binary logistic regression was used to analyze the relationship between affective symptoms and sleep quality. The final results showed that SAS (B = 0.068, p < 0.001, OR = 1.070, 95 % confidence interval (CI): 1.034–1.108), GDS (B = 0.112, p = 0.006, OR = 1.118, 95 % CI: 1.033–1.210) and age (B = 0.039, p = 0.005, OR = 1.040, 95 % CI: 1.012–1.068) were related to poor sleep quality. Table 3 presents the results.

**Table 2:** Comparison of the demographic, lifestyle and emotional symptom score of good sleepers and poor sleepers among the elderly in the community, based on the Pittsburgh Sleep Quality Index.

Variables	Poor sleeper (n = 249)	Good sleeper (n = 247)	X^2^ or T	p
Age				
55-59	32 (12.9)	53 (21.5)	9.135	0.104
60-64	59 (23.7)	60 (24.3)		
65-69	66 (26.5)	64 (25.9)		
70-74	47 (18.9)	41 (16.6)		
75-79	26 (10.4)	16 (6.5)		
80-	19 (7.6)	13 (5.3)		
Educational level				
illiterate	15 (6.0)	9 (3.6)	5.397	0.369
Primary school	18 (7.2)	23 (9.3)		
Junior high school	122 (49.0)	109 (44.1)		
High school	72 (28.9)	80 (32.4)		
Junior college	15 (6.0)	22 (8.9)		
University or above	7 (2.8)	4 (1.6)		
Male,n (%)	76 (36.5)	81 (37.3)	0.028	0.920
Smoker,n (%)	51 (20.5)	54 (21.9)	0.142	0.742
Drinker	61 (24.5)	53 (21.5)	0.648	0.456
Tea drinker	108 (43.4)	110 (44.5)	0.068	0.856
Physical exercise	148 (59.4)	165 (66.8)	2.888	0.095
Hypertension	111 (44.6)	108 (43.7)	0.037	0.857
Diabetes	43 (17.3)	34 (13.8)	1.161	0.322
SAS	33.70 ± 6.81	30.03 ± 6.16	6.034	<0.001*
GDS	3.53 ± 2.97	2.14 ± 2.70	5.329	<0.001*

Abbreviation: SAS, self-rating Anxiety Scale; GDS, Geriatric Depression Scale; *means p < 0.05.

**Table 3:** The influencing factors of poor sleep quality were investigated by binary logistics regression analysis.

Variables	B	S.E	Wald	df	p	OR	95%Confidence interval
SAS	0.068	0.018	14.632	1	<0.001*	1.070	1.034–1.108
GDS	0.112	0.040	7.680	1	0.006*	1.118	1.033–1.210
Age	0.039	0.014	8.025	1	0.005*	1.040	1.012–1.068
Males	0.023	0.211	0.012	1	0.913	1.023	0.677–1.548

Abbreviation: SAS, self-rating Anxiety Scale; GDS, Geriatric Depression Scale; *means p < 0.05.

### 4.3 The association between PSQI scores and GDS/SAS scores

By correlation analysis, controlling for age and sex, we found that the total score of GDS and anxiety was positively correlated with the total score of PSQI (p < 0.001), the correlation coefficients were 0.278 and 0.364, respectively (Table 4).

**Table 4:** Correlation analysis between SAS, GDS and PQSI (age and sex were controlled).

Variables	Mean (SD)	SAS	GDS	PQSI
SAS	31.84 ± 6.74	1	0.452**	0.364**
GDS	2.84 ± 2.93	0.452**	1	0.278**
PQSI	5.21 ± 3.79	0.364**	0.278**	1

Abbreviation: SAS, self-rating Anxiety Scale; GDS, Geriatric Depression Scale; PQSI, Pittsburgh Sleep Quality Index, *means p < 0.05; **means p < 0.001.

### 4.4 Association between sleep quality, emotion rating and brain structure

**Fig. 1:**
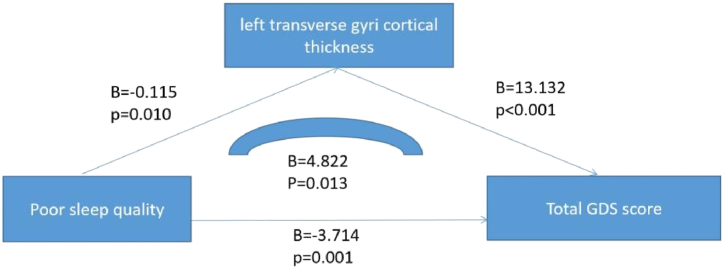
Linear regression analysis of mediation model.

Lastly, we explored the association of mood ratings with sleep-related brain regions, and 50 healthy individuals with no mild cognitive impairment and no dementia agreed to brain MR imaging. We divided them into poor sleepers (n = 21) and good sleepers (n = 29) according to their sleep quality. Those with poor sleep quality had greater left temporal transverse gyrus thickness and higher GDS and SAS scores (p < 0.05). However, there were no statistically significant differences between the two groups in age, education, sex, whole brain volume, left temporal transverse gyrus volume, right temporal transverse gyrus volume, and right temporal transverse gyrus thickness (p < 0.05) (Table 5). Linear regression analysis of the mediating model showed that poor sleep quality led to higher GDS scores, and the thickness of the left temporal transverse gyrus played a fully mediating role (B = 4.822, p = 0.013). The results are shown in Fig. 1. In contrast, there was no such association between SAS total score and thickness of the left transverse temporal gyrus (p > 0.05).

**Table 5:** Comparison of brain structure and emotional symptom between poor sleeper and good sleeper.

Variables	Poor sleeper (n = 21)	Good sleeper (n = 29)	X^2^ or T	p
Age, years	70.95 ± 7.65	69.45 ± 6.03	0.777	0.441
Education, years	7.70 ± 4.41	7.93 ± 4.21	−0.182	0.857
Male, n (%)	5 (23.8)	4 (13.8)	0.828	0.464
Whole brain volume, cm^3^	1386.94 ± 168.78	1334.91 ± 113.14	1.306	0.198
Left transverse temporal gyri volume, mm3	1.00 ± 0.18	0.94 ± 0.15	1.159	0.117
Right transverse temporal gyri volume, mm^3^	0.78 ± 0.13	0.75 ± 0.12	0.554	0.582
Left transverse temporal gyri thickness, mm^3^	2.31 ± 0.16	2.19 ± 0.14	2.698	0.010*
Right transverse temporal gyri thickness, mm^3^	2.29 ± 0.22	2.23 ± 0.02	1.072	0.289
SAS	27.37 ± 4.83	22.35 ± 3.61	3.495	0.001*
GDS	5.43 ± 4.37	1.71 ± 2.69	3.367	0.001*

Abbreviation: SAS, self-rating Anxiety Scale; GDS, Geriatric Depression Scale; *means p < 0.05.

## 5 Discussions

The relationship between sleep disorders and mood disorders is complex, and the pathways and mechanisms of their interaction are not well understood. Therefore, the purpose of this study was to explore the relationship between affective symptoms and sleep quality in Chinese community elderly, and to explore the potential mechanism of sleep quality decline affecting mood disorders. Finally, we observed several interesting findings:1) People with poor sleep quality had more symptoms of anxiety and depression; 2) The left transverse temporal gyrus cortex was thicker in patients with poor sleep quality; 3) Decreased sleep quality may affect depressive symptoms by affecting the thickness of the left temporal transverse gyrus cortex, which may play a complete mediating role in this process.

As the process of China's aging accelerates and life expectancy lengthens, of the total population, 17.88 % were 60 years of age or older [37]. On the other hand, the older population also faces a range of risks and challenges. Prior studies have shown that there were high rates of mental health disorders (typically depression and anxiety) in the older population throughout the world [ [38,39]]. In addition, this group is also more likely to experience decreased sleep quality [40]. However, relatively few studies have investigated the relationship between emotional symptoms and sleep quality in older adults. For example, in a study by Yu J et al., they found that symptoms of depression and anxiety in a group of older Asian adults were associated with sleep [41]. In Liao et al. 's study, they also found that poorer sleep quality was associated with higher levels of anxiety and depression [42]. In **Andruškienė J** et al. found that depressed mood is strongly associated with risky health behaviors, and women are more likely than men to have sleep disorders [43]**. In addition, some meta-analyses have also revealed some link between sleep disorders and mood disorders** [ [[44], [45], [46]]]**.** In this study, 496 community-dwelling older adults were included, and they were divided into two groups based on their sleep quality (good or poor). There were no significant differences in age, sex, education level, daily lifestyle, or chronic physical diseases between the two groups. However, individuals experiencing insufficient sleep reported a higher prevalence of symptoms related to anxiety and depression. Therefore, our findings are consistent.

Magnetic resonance imaging (MRI) is an effective tool for studying brain structure and function, helping to reveal possible mechanisms by which sleep disorders affect mood disorders. Accumulated evidence suggests that the somatosensory cortex plays a crucial role in all stages of emotional processing. Importantly, studies individuals with psychiatric disorders associated with abnormal emotion regulation, such as major depression, schizophrenia, post-traumatic stress disorder (PTSD), specific phobias, and obesity, have identified functional and structural alterations in the somatosensory cortex [47]. In addition, a systematic review has also shown that major depression is strongly associated with structural and functional changes in the temporal transverse gyrus [48]. Therefore, on the basis of synthesizing previous literatures and our previous studies, we took the volume of temporal gyrus and cortical thickness as the main observation indicators. After controlling for a range of variables, such as sex, age, education and total brain volume, we found that poor sleepers had a thicker left temporal cortex and higher anxiety and depression scores. Through the application of linear regression analysis on the mediating model, we discovered a significant association between GDS score and both sleep quality and the left temporal transverse gyrus cortical thickness. Furthermore, our findings indicate that the relationship between sleep quality and depression score is fully mediated by the thickness of the cortex. It should be noted was no observed correlation between anxiety score and temporal transverse gyrus cortical thickness.

As mentioned in the introduction, the transverse temporal gyrus is essential for pitch processing. At the same time, some research suggests that the transverse temporal gyrus may also be involved in mood symptoms such as depression. For instance, in the study of Barca ML et al., they found that higher depression scores were associated with thinner right right-transverse temporal cortex thickness in patients with dementia and depressive symptoms [35]. It is well known that physical and mental health can be influenced by family and community socioeconomic status (SES). In the study of Miller JG et al., they found a positive correlation between SES and the thickness of the left temporal transverse gyrus cortex [49]. In addition, Yrondi A et al. found that the thickness of the temporal transverse gyrus cortex was significantly higher in normal subjects than in depressed patients, however, this difference was no longer significant after patients received electroconvulsive Therapy (ECT) [50]. Therefore, we're basically in line with what other people were saying.

Interestingly, we demonstrate that the left transverse temporal gyrus cortical thickness may play an important regulatory role in the effects of sleep on mood. Topographic patterns of slow-wave activity (SWA) was negatively associated with daytime sleepiness, and in a study by Plante DT et al., they found that the temporal transverse cortex was decreased in patients with major depression [51]. Furthermore, in the study of Xu Y et al., they also found that sleep quality in patients with pulsating tinnitus (PT) was negatively correlated with anxiety scores and temporal transversal functional connectivity (FC) values [52]. Therefore, we speculate that cortical thickness in the transverse temporal cortex may be developed as a potential therapeutic target for depression. However, large follow-up studies or clinical intervention trials are needed to further test these hypotheses.

### 5.1 Limitations

There are some limitations to this study. Firstly, because this is only a cross-sectional study, it can't establish a cause-and-effect relationship between sleep quality and mood symptoms; Secondly, the magnetic resonance data is relatively small, which might cause some deviation to the research results. Finally, we lack an intervention to further test whether depressive symptoms could be treated by intervening in the thickness of the transverse temporal cortex.

## 6 Conclusions

Chinese community elders with poor sleep quality were likely to have more anxiety and depression symptoms, and had a thicker cortical thickness in the left temporal transitory cortex. Poor sleep quality may contribute to depressive symptoms by affecting the thickness of the left temporal transverse cortex. Therefore, we should monitor in the elderly population in order to mitigate their potential impact on sleep quality among older adults Furthermore, there exists a potential correlation between cortical in specific brain regions and anxiety, depression, as well as sleep quality. This association may serve as a promising biomarker for predicting the decline of sleep quality in older individuals.

## Funding

This rescarch was funded by the China Ministry of Science and Technology (STI2030-Major Projects-2022ZD0213100), the National Natural Science Foundation of China (82101564, 82001123, 82271607), the Feixiang Program of Shanghai Mental Health Center (2020-FX-03), Shanghai Clinical Research Center for Mental Health (19MC1911100), Shanghai brain health foundation（SHBHF2016001), Chinese Academy of Sciences (XDA12040101), Shanghai Clinical Research Center for Mental Health (SCRC-MH, 19MC1911100), and the Shanghai Science and Technology Committee (20Y11906800).

## Data availability statement

The authors do not have permission to share data.

## CRediT authorship contribution statement

**Wei Li:** Writing – original draft. **Lin Sun:** Writing – original draft. **Ling Yue:** Data curation. **Shifu Xiao:** Methodology, Funding acquisition.

## Declaration of competing interest

The authors declare that they have no known competing financial interests or personal relationships that could have appeared to influence the work reported in this paper.

## References

[1] Wolkove N., Elkholy O., Baltzan M., Palayew M. (2007). Sleep and aging: 1. Sleep disorders commonly found in older people. CMAJ (Can. Med. Assoc. J.).

[2] Gulia K.K., Kumar V.M. (2018). Sleep disorders in the elderly. psyg.

[3] Liu Y., Wheaton A.G., Chapman D.P., Cunningham T.J., Lu H., B J. (2016). Croft, prevalence of healthy sleep duration among adults--United States, 2014. MMWR Morb. Mortal. Wkly. Rep..

[4] Vézina-Im L.A., Morin C.M., Desroches S. (2021). Sleep, diet and physical activity among adults living with type 1 and type 2 diabetes. Can. J. Diabetes.

[5] Song Z., Jiang R., Li C., Jin F., Tao M. (2022). Menopausal symptoms and sleep quality in women aged 40-65 years. BioMed Res. Int..

[6] Hejazian S.M., Ahmadian E., Zununi Vahed S., Faraji Gogani L., Farnood F. (2021). The association of sleep quality and vitamin D levels in hemodialysis patients. BioMed Res. Int..

[7] Abdelaziz E.M., Elsharkawy N.B., Mohamed S.M. (2022). Health promoting lifestyle behaviors and sleep quality among Saudi postmenopausal women. Front. Public Health.

[8] Dong X., Wang Y., Chen Y., Wang X., Zhu J., Wang, Q N. (2018). Poor sleep quality and influencing factors among rural adults in Deqing, China. Sleep Breath..

[9] Yue Z., Zhang Y., Cheng X., Zhang J. (2022). Sleep quality among the elderly in 21st century shandong province, China: a ten-year comparative study. Int. J. Environ. Res. Publ. Health.

[10] Wu C.Y., Su T.P., Fang C.L., Yeh Chang M. (2012). Sleep quality among community-dwelling elderly people and its demographic, mental, and physical correlates. J. Chin. Med. Assoc..

[11] Ding L., Zhang L., Cui Y., Gong Q., Ma J., Wang Y. (2022). The association of sleep duration and quality with depressive symptoms in older Chinese women. PLoS One.

[12] Xiao S., Shi L., Zhang J., Li X., Lin H., Xue Y. (2023). The role of anxiety and depressive symptoms in mediating the relationship between subjective sleep quality and cognitive function among older adults in China. J. Affect. Disord..

[13] Hu Z., Zhu X., Kaminga A.C., Zhu T., Nie Y., Xu H. (2020). Association between poor sleep quality and depression symptoms among the elderly in nursing homes in Hunan province, China: a cross-sectional study. BMJ Open.

[14] Guo H., Zhang Y., Wang Z., Shen H. (2022). Sleep quality partially mediate the relationship between depressive symptoms and cognitive function in older Chinese: a longitudinal study across 10 years. Psychol. Res. Behav. Manag..

[15] Vazquez-Delgado E., Viaplana-Gutierrez M., Carlson C., Figueiredo R., Valmaseda-Castellon E. (2018). Sleep quality and psychosocial characteristics of patients with painful post-traumatic trigeminal neuropathies. Oral Surg Oral Med Oral Pathol Oral Radiol.

[16] Cheng W., Rolls E.T., Ruan H., Feng J. (2018). Functional connectivities in the brain that mediate the association between depressive problems and sleep quality. JAMA Psychiatr..

[17] Toschi N., Passamonti L., Bellesi M. (2021). Sleep quality relates to emotional reactivity via intracortical myelination. Sleep.

[18] Guadagni V., Burles F., Ferrara M., Iaria G. (2018). Sleep quality and its association with the insular cortex in emotional empathy. Eur. J. Neurosci..

[19] Zhang L., Bai Y., Cui X., Cao G., Li D., Yin H. (2022). Negative emotions and brain: egative emotions mediates the association between structural and functional variations in emotional-related brain regions and sleep quality. Sleep Med..

[20] Qi R., Cao Z., Surento W., Zhang L., Qiu L., Xia Z. (2022). RORA rs8042149 polymorphism moderates the association between PTSD symptom severity and transverse temporal gyrus thickness in Han Chinese adults who lost their only child. J. Affect. Disord..

[21] Zhang L., Cao G., Liu Z., Bai Y., Li D., Liu J., Yin H. (2022). The gray matter volume of bilateral inferior temporal gyrus in mediating the association between psychological stress and sleep quality among Chinese college students. Brain Imaging Behav.

[22] Kong L., Li H., Shu Y., Liu X., Li P., Li K., Xie W. (2021). Aberrant resting-state functional brain connectivity of insular subregions in obstructive sleep apnea. Front. Neurosci..

[23] Wang Y., Jiang P., Tang S., Lu L., Bu X., Zhang L. (2021). Left superior temporal sulcus morphometry mediates the impact of anxiety and depressive symptoms on sleep quality in healthy adults. Soc Cogn Affect Neurosci.

[24] Aydin B.K., Safali S., Aydin M., Egilmez U., Cebeci H., Çelik M. (2021). Does clozapine really affect bone mineral density? An experimental study. J. Orthop. Surg. Res..

[25] Li W., Yue L., Sun L., Xiao S. (2022). An increased aspartate to alanine aminotransferase ratio is associated with a higher risk of cognitive impairment. Front. Med..

[26] Farah N.M., Saw Yee T., Mohd Rasdi H.F. (2019). Self-reported sleep quality using the Malay version of the Pittsburgh sleep quality index (PSQI-M) in Malaysian adults. Int. J. Environ. Res. Publ. Health.

[27] Buysse D.J., Reynolds C.F., Monk T.H., Berman S.R., Kupfer D.J. (1989). The Pittsburgh Sleep Quality Index: a new instrument for psychiatric practice and research. Psychiatr. Res..

[28] Mollayeva T., Thurairajah P., Burton K., Mollayeva S., Shapiro C.M., Colantonio A. (2016). The Pittsburgh sleep quality index as a screening tool for sleep dysfunction in clinical and non-clinical samples: a systematic review and meta-analysis. Sleep Med. Rev..

[29] Shin C., Park M.H., Lee S.H., Ko Y.H., Kim Y.K., Han K.M. (2019). Usefulness of the 15-item geriatric depression scale (GDS-15) for classifying minor and major depressive disorders among community-dwelling elders. J. Affect. Disord..

[30] Jegede R.O. (1977). Psychometric attributes of the self-rating anxiety scale. Psychol. Rep..

[31] Li H., Jin D., Qiao F., Chen J., Gong J. (2016). Relationship between the Self-Rating Anxiety Scale score and the success rate of 64-slice computed tomography coronary angiography. Int J.Psychiatry Med.

[32] (2021). Management of osteoporosis in postmenopausal women: the 2021 position statement of the North American Menopause Society. Menopause.

[33] Jiang M., Wang Q., Finch T., She D., Zhou Y., Chung Y.F. (2022). Validity and reliability of the Chinese version of the normalization MeAsure development(NoMAD). BMC Health Serv. Res..

[34] Lin S., Yang Y., Qi Q., Wei L., Jing N., Jie Z. (2019). The beneficial effect of physical exercise on cognitive function in a non-dementia aging Chinese population. Front. Aging Neurosci..

[35] Barca M.L., Alnæs D., Engedal K., Persson K., Eldholm R.S., Siafarikas N. (2022). Brain morphometric correlates of depressive symptoms among patients with and without dementia. Dement. Geriatr. Cogn. Dis. Extra.

[36] Dale A.M., Fischl B., Sereno M.I. (1999). Cortical surface-based analysis. I. Segmentation and surface reconstruction. Neuroimage.

[37] Zhang L., Tao Y., Hou W., Niu H., Ma Z., Zheng Z. (2022). Seeking bridge symptoms of anxiety, depression, and sleep disturbance among the elderly during the lockdown of the COVID-19 pandemic-A network approach. Front Psychiatry.

[38] Curran E., Rosato M., Ferry F., Leavey G. (2020). Prevalence and factors associated with anxiety and depression in older adults: gender differences in psychosocial indicators. J. Affect. Disord..

[39] Jiménez A.L., Cruz-Gonzalez M., Calhoun T.F., Cohen L., Alegría M. (2022). Late life anxiety and depression symptoms, and suicidal behaviors in racial/ethnic minority older adults in community-based organizations and community clinics in the U.S, Cultur Divers Ethnic. Minor Psychol.

[40] Tian Y., Li L.M. (2017). [Epidemiological study of sleep disorder in the elderly]. Zhonghua Liuxingbingxue Zazhi.

[41] Yu J., Rawtaer I., Fam J., Jiang M.J., Feng L., Kua E.H. (2016). Sleep correlates of depression and anxiety in an elderly Asian population. Psychogeriatrics.

[42] Liao H., Liao S., Gao Y.J., Mu J.P., Wang X., Chen D.S. (2022). Correlation between sleep time, sleep quality, and emotional and cognitive function in the elderly. BioMed Res. Int..

[43] Andruškienė J., Podlipskytė A., Martinkėnas A., Varoneckas G. (2013). Depressive mood in association with sociodemographic, behavioral, self-perceived health, and coronary artery disease risk factors and sleep complaints. Medicina (Kaunas).

[44] Baglioni C., Nanovska S., Regen W., Spiegelhalder K., Feige B., Nissen C. (2016). Sleep and mental disorders: a meta-analysis of polysomnographic research. Psychol. Bull..

[45] Maghami M., Shariatpanahi S.P., Habibi D., Heidari-Beni M., Badihian N., Hosseini M. (2021). Sleep disorders during pregnancy and postpartum depression: a systematic review and meta-analysis. Int. J. Dev. Neurosci..

[46] Becker N.B., Jesus S.N., João K., Viseu J.N., Martins R.I.S. (2017). Depression and sleep quality in older adults: a meta-analysis. Psychol. Health Med..

[47] Kropf E., Syan S.K., Minuzzi L., Frey B.N. (2019). From anatomy to function: the role of the somatosensory cortex in emotional regulation. Braz J. Psychiatry.

[48] Brenner A.M., Claudino F.C.A., Burin L.M., Scheibe V.M., Padilha B.L., de Souza G.R. (2022). Structural magnetic resonance imaging findings in severe mental disorders adult inpatients: a systematic review. Psychiatry Res. Neuroimaging..

[49] Miller J.G., López V., Buthmann J.L., Garcia J.M., Gotlib I.H. (2022). A social gradient of cortical thickness in adolescence: relationships with neighborhood socioeconomic disadvantage, family socioeconomic status, and depressive symptoms. Biol Psychiatry. Glob Open Sci..

[50] Yrondi A., Nemmi F., Billoux S., Giron A., Sporer M., Taib S. (2019). Grey Matter changes in treatment-resistant depression during electroconvulsive therapy. J. Affect. Disord..

[51] Plante D.T., Cook J.D., Barbosa L.S., Goldstein M.R., Prairie M.L., Smith R.F. (2019). Establishing the objective sleep phenotype in hypersomnolence disorder with and without comorbid major depression. Sleep.

[52] Xu Y., Shi Y., Yao J., Yang H., Ding Z., Chen Q.Q. (2019). Altered brain functional connectivity and correlation with psychological status in patients with unilateral pulsatile tinnitus. Neurosci. Lett..

